# Myocardial Mitochondria at the Intersection of Health and Disease

**Published:** 2013-03-16

**Authors:** J. G Edwards

**Affiliations:** Department of Physiology, New York Medical College, Valhalla NY, USA

The air that we breathe and the food that we eat reach their confluency in the mitochondria to derive the energy that the cell is dependent upon. The heart is the most aerobic of organs and has little anaerobic reserve compared to the constant demands placed upon it. At rest the arterial-venous oxygen extraction from the blood is the greatest from the heart and this only goes up with exercise. Common to all cell types residing in the heart is the need for energy and mitochondrial dysfunction is a significant contributor to cell death across the spectrum of cardiac disorders. Mitochondrial failure within any one cell type creates varying complications that eventually manifest as heart failure.

The field of mitochondrial biology has under gone several paradigms shifts since the early nineties. The pioneering work of Krebs, Chance, and others had brought the field to a plateau with the focus on ATP generation. It was not until the implications of mitochondria’s role in apoptosis and the cell’s health, was there a strong resurgence of interest in mitochondrial biology [1,2]. Since then, a second major road of significance, dug predominantly by D. Wallace, has been in identifying the participation of mitochondria DNA (mtDNA) alterations and damage as a common pathology in unrelated symptoms [3,4]. And third avenue, that mitochondria are not just the isolated organelles pictured in electron micrographs, but dynamic entities that undergo significant morphological changes as a course of their normal function [5].

The mitochondrial genome is a circular double-stranded DNA of more than 16 Kb in humans. It codes for 37 genes including 13 of the more than 1000 proteins indigenous to the mammalian mitochondria. Mitochondrial disorders are a heterogeneous group of diseases that may be characterized by maternal inheritance, heteroplasmy, and threshold effect. Mitochondrial dysfunction and mtDNA damage has been reported in diabetes, alcoholism, cancer, skeletal muscle disorders, and neurodegenerative diseases such as Barth Syndrome, MELAS, ALS, or LHON [6–9]. As one example, several mtDNA mutations have been identified that represent a high risk for the development of diabetes [10–13]. Mutations may take the form of deletions, rearrangements, or missense mutations that interfere with protein synthesis. The etiology for the accumulation of mtDNA mutations and deletions is not completely understood [14–16].

Separate from inborn errors, mutations and deletions of mitochondrial DNA (mtDNA) accumulate as a function of the aging process or from environmental influences and thought responsible for the decline in mitochondrial function [14,17]. Indeed the “mitochondrial theory of aging” is centered on the accumulation of mtDNA mutations and an increase in mitochondrial oxidative stress, creating a “vicious” circle that accelerates this process. Mitochondrial DNA is thought to be at greater risk for oxidant-induced damage due to its close proximity to the electron transport chain and high levels of superoxide production. Also, unlike genomic DNA, mtDNA contains little intron DNA which may serve to absorb damage from chronically elevated oxidative stress. In postmitotic cells, the mitochondrial genome continues to replicate about once a month [18]. Oxidant-induced mtDNA mutations that are not corrected by mitochondrial DNA repair mechanisms are fixed in the mitochondrial genome.

Mitochondrial dependent ROS (mtROS) generation has been accepted as the singular cause of mtDNA damage in different pathophysiologic states [19]. However, antioxidant therapy studies have yielded results that range from disappointing to a potentially detrimental effect of antioxidants [20–23]. Other approaches that raise or lower mitochondrial antioxidant capacity have also yielded conflicting results [24,25].

Historically, ROS has been viewed as a waste byproduct of aerobic metabolism. However the cell’s ability to generate specific different oxidant species indicates a useful purpose for normal cell function [26,27]. More recently, this has taken the concept of compartmentalization of signaling; by physical separation of ROS production as well as activation of distinct enzyme complexes [26,27]. Several lines of evidence point towards increased mtROS as a significant cause of mitochondrial dysfunction [28–34].

A limitation to some studies is that they examined mtROS changes only as an early event. Others that examined an extended timeframe point to a more complex interaction of ROS within the mitochondria and suggest that alternative pathways may mediate the effect of ROS on mitochondrial function and mtDNA integrity [35–37]. Mitochondria have both endogenous oxidant buffers and mtDNA repair capability but this failure suggests that there may be limits to functional recovery. It may be that the pathology generates a transient signal either internal or external to the mitochondria that is prolonged by activation of pathways that exacerbate the initial insult. Going forward, work to differentiate the different oxidant species and their sources in the normal and diseased heart will clarify their respective roles.

The concept that increased mtDNA mutation rates leads to a vicious cycle of increased oxidative stress has been challenged by investigations using transgenic mice that express a cardiac-specific “proof-reading deficient” mitochondrial DNA polymerase (mtDNA-Pol^def^). In those studies cardiac cells accumulated mtDNA mutations at a rate of more than 20 fold compared to controls, demonstrated increased apoptosis, and presented with significant heart failure [38,39]. Despite this, there was not a significant change in mitochondrial function; the P/O ratio and respiratory control index were similar in mtDNA-Pol^def^ and controls. Significantly, markers for ROS did not increase suggesting that oxidative stress was not an obligate mediator of mtDNA mutations. This model suggests that any increase in mtDNA mutations may serve as a signal for the initiation of apoptosis. Further, Herlein et al have argued that in mild diabetes or prediabetes, mitochondrial superoxide may not be elevated in contrast to its decided presence in more severe diabetic states [31]. More recently our investigations suggest that separate from a direct effect of mtROS on mtDNA, mitochondrial topoisomerase dysfunction increased mtDNA strand breakage [37,40]. Collectively these studies suggest that more than just mtROS promotes mtDNA damage leading to mitochondrial and cellular degradation within the heart. And it remains that preservation and protection of mtDNA becomes a focal point for novel clinical strategies.

Mitochondria are dynamic organelles and the fission/fusion processes have a significant role in the myocardium. The familiar pictures from electron microscopy showing distinct mitochondria interspersed amongst the sarcomere are somewhat misleading. It portrays the mitochondria as individual entities, but we now known they may form reticulated networks [5]. This dynamic exchange allows for sharing of mitochondrial contents. If sharing did not occur then it would be necessary for each mitochondrion to coordinate with the nucleus to import of all the proteins it could not be synthesized. Sharing also allows for the dilution of mtDNA mutations that would directly interfere with respiratory function; permitting greater mutational loads to be carried.

Importantly, sharing may allow for segregation of dysfunction mitochondria as an early step towards autophagy or apoptosis [41], increased fission resulting in smaller more numerous mitochondria has been associated with elevated caspase activity, an initial step in apoptosis [42]. To date several proteins critical for fusion and fission processes have been identified including Mfn1, Mfn2, OPA1, Drp1, & PINK1. Although mutations of OPA1 and Mfn2 are mostly associated with the neuropathy Charcot-Marie-Tooth disease, they also have a role in myocardial mitochondrial function [43,44]. Inhibition of Drp1 appeared to protect mitochondria during an ischemia/reperfusion challenge suggesting that control of mitochondrial morphology is clinically relevant [45]. Beyond the gain of function and loss of function experimental paradigms, the field is still developing the tools to study the role of fusion and fission in myocardial mitochondria. We know that both processes are essential for maintenance of the phenotype, but specific perturbations associated with different pathologies remain to be more fully explored.

Cardiovascular disease has a higher incidence of mortality in patients with diabetics, alcoholism, cancer, and survivors of cancer treatment compared to the general population. The continuously beating heart is the most metabolically active organ in the body. This would not be possible without the continuous support from the mitochondria. This view developed in the early part of the 20^th^ century brought the field to a plateau of understanding. Apoptosis was originally described as a developmental step but its role in the maintenance of the phenotype cleared the path for mitochondria’s participation. Beyond this has come the recognition of the many roles mitochondria has beyond ATP regeneration and their vulnerability in cardiac pathology. As we learn more, viable new clinical strategies will hopefully become apparent to protect mitochondrial function in the heart.
